# Wernicke's Encephalopathy: An Unusual Consequence of the Acquired Immune Deficiency Syndrome—Case Report and Literature Review

**DOI:** 10.1155/2013/709474

**Published:** 2013-07-09

**Authors:** Timothy R. Larsen, Dritan Dragu, Michael Williams

**Affiliations:** Department of Internal Medicine, Providence Hospital and Medical Centers, 16001 West Nine Mile Road, Southfield, MI 48075, USA

## Abstract

*Introduction.* Wernicke's encephalopathy is a well-described syndrome characterized by the classic triad of confusion, ataxia, and ophthalmoplegia. Wernicke's encephalopathy results from thiamine (vitamin B1) deficiency. Common causes include alcoholism and gastric disorders. Wernicke's has been described in patients with acquired immune deficiency syndrome (AIDS); however, given these patients' immunosuppressed state, the diagnosis of Wernicke's encephalopathy is not apparent. *Case Presentation.* A 31-year-old previously healthy male presented to the ER complaining of progressive dyspnea. Workup revealed HIV/AIDS and PCP pneumonia. He was treated and improved. On day 14 he became confused and developed nystagmus and ataxia. Considering his immunocompromised state, infectious and neoplastic etiologies topped the differential diagnosis. CT head was negative. Lumbar puncture was unremarkable. Brain MRI revealed increased T2 signal in the medial thalamus bilaterally. Intravenous thiamine was administered resulting in resolution of symptoms. *Discussion.* The classic triad of Wernicke's encephalopathy occurs in 10% of cases. When immunosuppressed patients develop acute neurologic symptoms infectious or neoplastic etiologies must be excluded. However, given the relative safety of thiamine supplementation, there should be a low threshold for initiating therapy in order to reverse the symptoms and prevent progression to Korsakoff dementia, which is permanent.

## 1. Introduction

Wernicke's encephalopathy (WE) is a well-described syndrome characterized by the classic triad of confusion, ataxia, and ophthalmoplegia. WE results from a deficiency of thiamine (vitamin B1). In the United States common causes include alcoholism, repetitive vomiting, and gastric disorders including chronic gastritis, Crohn's disease, or after bariatric surgery [1]. WE has also been described in patients with acquired immune deficiency syndrome (AIDS); however, given these patients' susceptibility to central nervous system infections and tumors (such as lymphoma), the diagnosis of WE is often not apparent. 

Recognizing WE in a patient with AIDS poses a special challenge for physicians. In the presence of AIDS, WE often does not present in its most classic form. Additionally, the neurologic symptoms encountered in WE can significantly overlap with other, more common conditions. The diagnosis of WE, therefore, is often not made until autopsy. Herein, we report a patient with newly diagnosed HIV/AIDS hospitalized with an opportunistic infection who developed reversible neurologic symptoms consistent with WE.

## 2. Case Presentation

A 31-year-old male presented to the emergency room complaining of progressive dyspnea and productive cough over the course of 4 months. He also noted decreased appetite with a 50-pound weight loss over the past 5 months. He had no significant past medical or surgical history and was not taking any medications. His family history was unremarkable. He quit smoking tobacco two months ago due to worsening respiratory status; he rarely used alcohol and never used recreational drugs. He was unmarried and in a long-term heterosexual relationship and denied multiple sexual partners. He was currently working in an automobile parts factory as a janitor. After high school he had enlisted in the army and served for four years; at that time he was stationed in Hawaii. While in Hawaii he visited a single prostitute on several occasions.

Initial vital signs revealed temperature was 99.9 F, blood pressure 128/83 mmHg, pulse 134 beats per minute, and respiratory rate 22 breaths/minute; oxygen saturation was 92% on room air. He was in moderate respiratory distress, yet was awake, alert, and cooperative. Extraocular muscles were intact; there was no nystagmus or scleral icterus. Pupils were equal, round, and reactive to light. He had thrush under his tongue and on the palate. There was no palpable lymphadenopathy. Heart rate was regular with no murmurs, rubs, or gallops by auscultation. There were no palpable thrills. He had equal chest rise bilaterally. Breath sounds were diminished throughout, with rales and dullness to percussion in the lower lung fields bilaterally. Sensory and motor examination was grossly intact.

**Figure 1:**
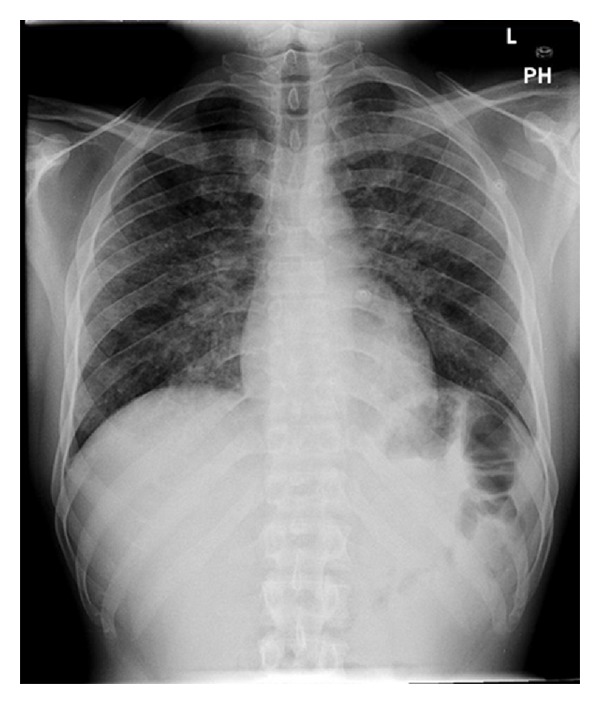
Upright posterior-anterior projection chest X-ray demonstrating diffuse reticular-nodular opacities in both lungs.

**Figure 2:**
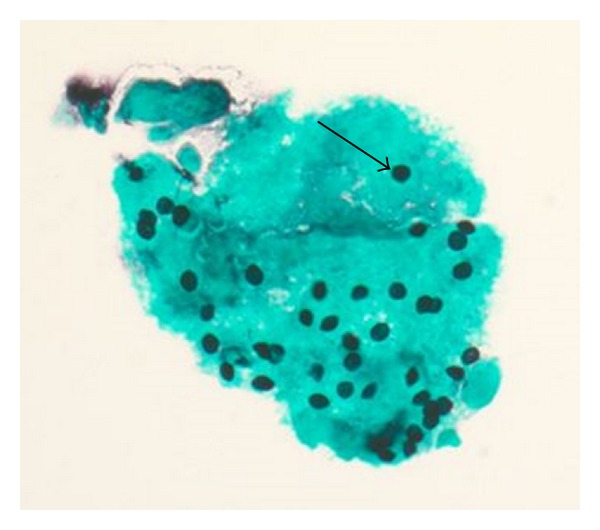
Grocott's methenamine silver (GMS) stain of bronchoalveolar lavage specimen demonstrating black staining round cysts (arrow) in the alveolar exudate diagnostic of *Pneumocystis jiroveci *pneumonia.

Laboratory data revealed white blood count 13,900/mcL, hemoglobin 7.2 g/dL, platelets 370,000/mcL, sodium 131 mmol/L potassium 5.8 mmol/L, chloride 99 mmol/L, carbon dioxide 17 mmol/L, blood urea nitrogen 155 mg/dL, and serum creatinine 8.9 mg/dL. Arterial blood gases showed a pH of 7.39, pCO_2_ 32.2 mmHg, bicarbonate 19.1 mmol/L, and pO_2_ 74.5 mmHg on 3 L nasal cannula. Chest X-ray showed diffuse bilateral reticulonodular opacities (Figure 1). He was found to be HIV positive with CD4 count of <5 cells/cc and viral load of 1,180,000 copies. Bronchoalveolar lavage was preformed; Grocott's methenamine silver stain of the washings demonstrated *Pneumocystis jiroveci *(Figure 2). He received antibiotics and steroids with gradual improvement.

**Figure 3:**
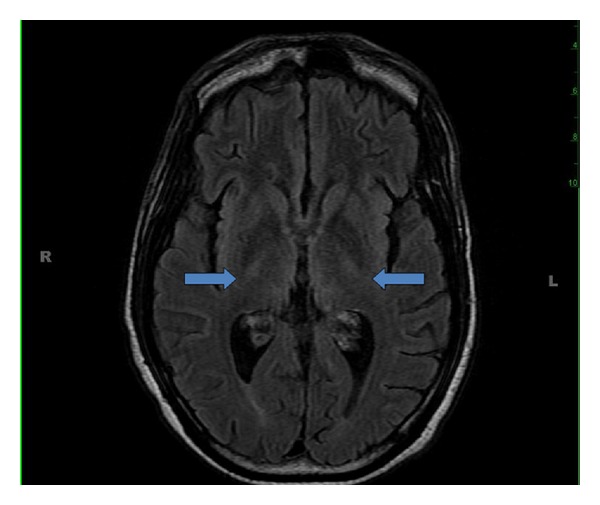
MRI demonstrating slight increase in T2 signal in the medial bilateral thalamus (arrows).

On hospital day 14, he became confused and acutely developed nystagmus with both a horizontal and prominent vertical component. He was also found to have ataxia with finger to nose testing and difficulty sitting up without support. Considering his immunosuppressed state, infectious and neoplastic etiologies were the primary diagnostic considerations. Lumbar puncture revealed clear CSF, normal opening pressure, and normal cell counts. Polymerase chain reactions for Epstein-Barr virus, Herpes simplex virus, cytomegalovirus, and John Cunningham (JC) virus were negative. Nontreponemal serological screening for syphilis (VDRL), toxoplasmosis antibody, and *Cryptococcus* antigen titers were negative. CT of the head did not show any intracranial mass, hemorrhage, or other acute findings. MRI of the brain revealed a slight increase in T2 signal within the medial aspect of thalamus bilaterally (Figure 3). After excluding infection and neoplasm, the clinical diagnosis of Wernicke's encephalopathy was made. Intravenous thiamine was administered resulting in resolution of symptoms, which confirmed the diagnosis.

## 3. Discussion

Wernicke's encephalopathy results from a deficiency of thiamine (vitamin B1), historically associated with alcoholism. Over the past three decades there have been a number of reports that find WE in other disease states not associated with alcohol. Therefore, thiamine deficiency should be born in mind in all patients with evidence of not only alcoholism, but also AIDS, malignant tumors, chronic inflammation, malnutrition, malabsorption, and any other severe diseases.

WE is largely a clinical diagnosis based on physical exam findings along with supportive imaging. Classically WE presents with an acute onset of confusion, ocular abnormalities, and ataxia. It is characterized by disorientation, impairment of memory, nystagmus, extraocular palsies, miotic pupils, slow pupillary reflexes, and cerebellar imbalance. Peripheral neuropathy and reduced tendon reflexes, seizures, nausea, and vomiting can also occur [2, 3]. Typically, once present, symptoms progress rapidly leading to death within a week if treatment is not instituted.

According to the 2010 European Federation of Neurological Societies (EFNS) guidelines, the clinical diagnosis of WE in an alcoholic patient requires two of the following: (i) dietary deficiencies (ii) eye signs, (iii) cerebellar dysfunction, and (iv) either an altered mental state or mild memory impairment. When secondary to long standing alcoholism, WE often develops gradually taking an indolent course, which is contrasted by our case where WE developed rapidly (which is characteristic of nonalcoholic WE) [3]. Our patient rapidly developed all three classic signs of WE, which are seen in combination only in 8% of the cases [3]. 

The first reported case of HIV/AIDS-associated WE was described by Foresti and Confalonieri in 1987 [4]. Since then only 11 individual cases of nonalcoholic, HIV-related WE have been reported in the English literature (Table 1) [4–10]. All patients died shortly after the development of neurological symptoms. In five of the cases, acute WE was determined to be the direct cause of death [7]. Prior to the presented case, WE in only one of the reported cases was considered and treatment initiated antemortem. Autopsy typically demonstrates patchy spongiosis, neuronal shrinkage, acute neuronal necrosis, capillary proliferation in the mamillary bodies and periaqueductal tissue, petechial hemorrhages, and glial proliferation [6].

The prevalence of thiamine deficiency (which directly causes WE) in the AIDS population has been estimated at around 23% [11, 12]. Butterworth et al. demonstrated biochemically confirmed thiamine deficiency in 9 out of 39 patients with AIDS; all patients had no prior history of alcohol abuse or clinical signs of WE [11]. Additionally, in an autopsy series including 400 patients diagnosed with AIDS, Boldorini et al. reported pathologic changes in the central nervous system consistent with WE in up to 10% of cases [12]. Opportunistic infections were four times more common, occurring in 40% of cases. 

The overall catabolic state associated with AIDS results in malnourishment which is the presumed pathophysiologic mechanism responsible for thiamine deficiency and subsequent WE. Malnutrition is a well-established risk factor for WE regardless of underlying etiology [8]. 

Thiamine has a key role in glucose metabolism and thus energy production. In Kreb's cycle, thiamine mediates transformation of pyruvate to acetyl-CoA and transformation of *α*-ketoglutarate to succinyl-CoA. With thiamine deficiency, pyruvate accumulates which is converted to lactate as cells switch from aerobic to less efficient anaerobic metabolism. Lactate accumulation results in metabolic acidosis with resultant disturbances in systemic and cerebral metabolism. Within 1-2 weeks after thiamine depletion there are regions of cerebral hypoperfusion and a functional disruption in the blood-brain barrier. A healthy well-nourished adult has approximately a 2-month store of thiamine [13].

Dementia is a common complication of AIDS and may afflict as many as 60% of patients. While dementia may result from a variety of etiologies (including opportunistic infections, CNS lymphomas, and direct cerebral HIV infection), WE should be considered in the presence of mild to moderate cognitive impairment [14]. Central nervous system infection or neoplasm must be excluded in the immunosuppressed population when considering WE. MRI is the imaging modality of choice to support the diagnosis of WE. MRI has a sensitivity and specificity of 53% and 93%, respectively, with a positive predictive value of 89% [3]. 

Given the overall safety of thiamine supplementation, there should be a low threshold for initiating therapy in order to reverse the symptoms of WE and prevent progression to Korsakoff dementia, which is irreversible. Butterworth et al. advocate for thiamine supplementation in all patients diagnosed with AIDS [11]. For treatment of symptomatic WE a single double-blinded randomized trial (conducted in alcoholics) identified 200 mg of thiamine IM daily for two days as the preferred dose to reverse cognitive impairment [3]. EFNS recommends 200 mg of thiamine thrice daily, preferably intravenously, before any carbohydrate is given, to be continued until there is no further improvement in signs and symptoms. Our case demonstrates the reversibility of the neurologic deficits typical of WE with thiamine supplementation. We suggest thiamine supplementation begins at the time of diagnosis of AIDS. 

## 4. Conclusion

Considering the confirmed neuropathologic evidence of WE in the AIDS, documented thiamine deficiency in these patients, and variability in clinical presentation of WE, we recommend thiamine supplementation in all newly diagnosed cases of AIDS.

## Figures and Tables

**Table 1:** Reported cases of Wernicke's encephalopathy.

Author	Year	Age	Antemortem diagnosis	Initial complaint	Other findings	Alcoholism
Foresti	1987	26	No	NR	Malnutrition	No
Davtyan	1987	46	No	Diarrhea, cough, weakness	Seizure, GI bleeding	No
Soffer	1989	40	Yes	Fever and cough	PCP pneumonia, *Candida * meningitis	No
Schwenk	1990	36	No	GI bleeding	Tuberculosis, lymphoma	No
Schwenk	1990	39	No	Fever, weight loss, cognitive impairment	Pneumonia	No
Monatine	1993	41	No	Weight loss, diarrhea, blurry vision	MAC, candidiasis	No
Burdge	1995	33	No	Fatigue, unsteady gait, slurred speech	PCP, herpetic esophagitis	No
Alcaide	2003	37	No	Altered mental status, unsteady gait, slurred speech	Syphilis	No
Gui	2006	59	No	Abdominal pain, jaundice, vomiting	Malnutrition	No
Gui	2006	73	No	Altered mental status	Malnutrition	No
Gui	2006	33	No	Jaundice, vomiting	Malnutrition	No
Larsen	2013	31	Yes	Dyspnea, cough, weight loss	PCP pneumonia	No

NR: not reported; GI: gastrointestinal; PCP: pneumocystis pneumonia; MAC: mycobacterium avium complex.
